# Corrosion Activity of Ultrafine-Grained Pure Magnesium and ZK60 Magnesium Alloy in Phosphate Buffered Saline Solution

**DOI:** 10.3390/ma17112726

**Published:** 2024-06-04

**Authors:** Stella Diederichs, Dayan Nugmanov, Yulia Ivanisenko, Eberhard Kerscher

**Affiliations:** 1 Working Group Materials Testing (AWP), RPTU Kaiserslautern-Landau, Gottlieb-Daimler-Straße, 67663 Kaiserslautern, Germany; 2 Institute of Nanotechnology, Karlsruhe Institute of Technology (KIT), Hermann-von-Helmholtz-Platz 1, 76344 Eggenstein-Leopoldshafen, Germany

**Keywords:** magnesium corrosion, grain refinement, biodegradable metals

## Abstract

The magnesium alloy ZK60 is a promising candidate as a material for biodegradable implants. One of the most important factors for biodegradable implants is the modification of their corrosion behavior to match the requirements for the healing bone or tissue. The corrosion behavior can be influenced by different factors, among them the grain size, which can be changed by severe plastic deformation processes such as High Pressure Torsion Extrusion (HPTE). This study focuses on the corrosion behavior of samples of pure magnesium and ZK60 before and after HPTE, and the influence of the microstructure on the corrosion activity. The samples are subjected to immersion tests in phosphate buffered saline solution (PBS). The corrosion activity is defined by the emerging hydrogen volume from the corrosion process which is collected and by subsequently observing the resulting sample surfaces. The findings of this study suggest that pure magnesium shows lower corrosion activities than ZK60 and that HPTE processing leads to higher corrosion activities in PBS.

## 1. Introduction

Magnesium alloys, such as ZK60, are promising materials for biodegradable implants [1,2]. The well-known pronounced corrosion activity of magnesium is used in this case to obtain implants that degrade in the body after they have served their purpose [2]. Advantages range from the mechanical properties of magnesium that are in the order of magnitude of bone and can so prevent stress shielding through its good biocompatibility to the lack of a need for removal surgery [1,2,3,4,5,6].

To apply magnesium alloys as biodegradable implants, it is crucial that the corrosion activity and thus the corrosion rate of the material is adapted to the healing process of the bone and/or surrounding tissue [7]. Consequences of the material corroding too fast are, e.g., subcutaneous gas pockets which are detrimental for the healing process or the premature loss of its mechanical integrity [1,5,8].

The corrosion activity of magnesium depends on many different variables, such as the alloy composition, the microstructure of the sample and the environment to which the sample is exposed.

Alloying of magnesium with other metals can increase or decrease the corrosion rate depending on the alloying elements and the amount of the elements added to the alloy [3]. The alloying elements can form secondary phases which are in most cases more noble than magnesium and act as local cathodes in the material. In the case that these secondary phases are finely dispersed in the material or form continuously along the grain boundaries, they can have positive effects on the corrosion resistance [9,10]. However, if these secondary phases are rather localized, this can lead to pitting corrosion and a quicker corrosion of the matrix material [9,11].

Other influencing factors of the material on the corrosion activity are, among others, the grain size [10,12,13,14,15] and the dislocation density [4,16]. The influence of the grain size on the corrosion activity is widely discussed in the literature, with some authors reporting an increase in the corrosion activity with decreasing grain size [10,13] and other authors reporting the opposite [14,15]. A smaller grain size leads to a larger volume fraction of grain boundaries in the material. Due to the mismatch of the crystals at grain boundaries, they can act as anodic sites where corrosion activity can rise [4]. On the other hand, a larger amount of grain boundaries can help to improve the mismatch of the corrosion surface layer with the material and thus decrease the corrosion rate [14].

A decrease in grain size is often achieved by severe plastic deformation (SPD) processes. These processes can introduce a lot of strain and dislocations into the material. Dislocations, being defects in the material, also lead to weaker bonds between neighboring atoms [4]. Song et al. report that the higher dislocation density after SPD leads to higher corrosion rates because of this reason [10].

Besides the material and its microstructure, the environment which the material is exposed to has a great influence on the corrosion behavior. In particular, the chloride concentration and the pH of aqueous solutions play important roles [17]. Zhao et al. showed that higher chloride ion concentrations and lower pH values of the environment lead to higher corrosion rates [18]. In the human body, an implant is exposed to body fluids, such as blood. Blood has a pH value of 7.4 and contains, besides water and organic cells, also electrolytes such as chloride, sodium, potassium, magnesium, bicarbonate and phosphate [19]. The average chloride concentration in human blood is around 0.1 M [20].

The corrosion rate of magnesium alloys can be modified by different techniques. Besides alloying and surface coatings [4], a modification of the microstructure towards finer grains has also proven to be beneficial [7,14,21]. Such a grain refinement can be achieved for example by SPD processes, which lead to a grain refinement without changing the alloy composition, such as High-Pressure Torsion Extrusion (HPTE). In the HPTE process, a sample is submitted to an extrusion process and a superimposed torsional deformation at a given temperature. The combination of these two leads to a high strain in the material after a single pass [22]. Compared to other SPD-based methods such as high pressure torsion [23] or multi-directional forging [24], HPTE has several advantages for processing bulk nanocrystalline materials in amounts suitable for industrial applications. Furthermore, it can be combined with the Conform method to process ultrafine-grained (UFG) rods with practically unlimited length [25]. This option is very important for the industrial application of the HPTE process for the manufacturing of biomedical implants and stents, and in many other branches of industry, for example, energy (high-strength electric cables), transportation, aerospace, etc.

In the present study the corrosion behavior of pure magnesium and ZK60 samples was investigated. Pure magnesium was used for comparison to evaluate the influence of alloying elements and second phase particles on the corrosion behavior of UFG magnesium. The ZK60 alloy exhibits good biocompatibility. Furthermore, the main alloying elements, Mg, Zn and Zr, are biocompatible, and Mg and Zn are essential minerals demanded by the human body. Alloying of pure magnesium can significantly increase mechanical strength by more than 50% [26]. However, alloying reduces the corrosion properties of magnesium. It is therefore important to investigate the effect of HPTE processing on the corrosion behavior of the ZK60 alloy in comparison with pure magnesium.

## 2. Materials and Methods

### 2.1. Materials

Pure magnesium (99.95% purity), supplied by Material, Technologie & Kristalle GmbH (Juelich, Germany), and the Mg alloy ZK60, supplied by MagIC—Magnesium Innovations Center, Helmholtz Center Hereon (Geesthacht, Germany), were investigated in this study. The chemical composition of ZK60 is described in Table 1.

The as-received pure magnesium material was annealed for 30 min at 500 °C and then furnace cooled. After annealing, samples were extruded at 200 °C with an extrusion ratio of 3:1 and subsequently processed by HPTE at 180 °C with a translational velocity of 6 mm/min (v6) and a rotational velocity of 0.6 rpm (ω0.6). During preliminary experiments, it was established that 180 °C is the optimal processing temperature for HPTE processing of pure Mg. Processing at lower temperatures led to the formation of surface cracks, and processing at higher temperatures resulted in larger mean grain sizes.

The HPTE equipment and procedure used in this study were described in [22], with the only difference that an octagonal die, specially designed for processing of materials with low deformability, was used.

Samples of the ZK60 alloy were investigated in the as-received state and after HPTE. The as-received samples were obtained by extrusion at 300 °C with an extrusion ratio of 17.4:1 (henceforth referred to as “as-extruded state”). Some as-extruded samples were processed by HPTE at 150 °C and 250 °C using the same translational and rotational velocities as those used for pure magnesium. Processing temperatures were chosen for same reasons as for pure magnesium. In particular, preliminary experiments demonstrated that HPTE of the ZK60 alloy at temperatures lower than 150 °C led to surface cracking. An additional processing at 250 °C was performed because different deformation aging kinetics and consequently different volume fractions of hardening particles were expected in comparison with the state after HPTE at 150 °C. In total, five different material states were thus produced as shown in Table 2.

### 2.2. Sample Preparation

Samples for corrosion testing were cut from the initial rods perpendicular to the extrusion direction and embedded in cold mounting resin. Subsequently, the samples were ground on a Tegramin-25 grinding and polishing machine from Struers using P1200, P2000 and P4000 grinding papers each for 90 s at a force of 20 N with water as the lubricant. The following polishing process on the same machine started with diamond suspensions of 9 µm, 3 µm and 1 µm with a mixture of glycerol and ethanol (1:3) as lubricant. Each of these three steps was carried out for 5 min at a force of 15 N. A polishing step with a mixture of diamond suspension of 0.05 µm and Nital 3% (49:1) for 5 min with a force of 25 N and again glycerol and ethanol as lubricant followed. Afterwards, the samples were polished further on a Qpol Vibro from QATM (Mammelzen, Germany) for 20 min at 60% of the maximum intensity and a frequency of 90 Hz using an anhydrous alumina suspension. Following this step, the samples’ surfaces were freed from residual alumina using a suspension of alumina in demineralized water for 10 s at a force of 5 N on the polishing machine. In a last step, the samples were cleaned using ethanol.

Preparation of samples for SEM observations involved a standard electro-polishing procedure using a Tenupol-5 twinjet polisher and a Struers electrolyte A2. To ensure optimal results, a final polishing step was performed using the GATAN PIPS system to eliminate surface oxide layer.

### 2.3. SEM and EBSD

The microstructure of the samples was examined via Electron Backscatter Diffraction (EBSD) technique. This analysis was conducted using a Zeiss Auriga 60 scanning electron microscope (SEM) (Oberkochen, Germany) operating at 20 kV and equipped with an EDAX-TSL EBSD system.

EBSD patterns were collected using APEX EBSD EDAX 2.5 software (EDAX Inc., Draper, UT, USA). The scanning areas had different dimensions: 1.3 mm × 0.6 mm for the annealed sample, 210 µm × 150 µm after extrusion and 150 µm × 100 µm after HPTE. Orientation imaging microscopy (OIM) maps were acquired from longitudinal sample cross-sections. The data were collected at the middle radius (2.7 mm from the sample axis). The scanning step was set at 1 µm for the initial state and the central part of the deformed samples. In the middle radius region of the as-extruded and HPTE-processed samples, the scanning step was reduced to 0.05 µm.

The obtained OIM maps were analyzed using OIM TSL 8.6 EDAX software. To minimize misindexing errors, nine Kikuchi bands were employed for indexing. In order to ensure the reliability of the EBSD data, grains consisting of three or fewer pixels were excluded from the maps. This was achieved using the grain-dilation and neighbor orientation correlation features of the TSL 8.6 software.

A 15° criterion was utilized to distinguish between low-angle boundaries (LABs) and high-angle boundaries (HABs). The grain size (D) was calculated using the equivalent diameter method. Two boundary misorientation threshold values, 2° and 15°, were used to define the grain. The corresponding mean grain sizes were denoted as D2 and D15. For statistical analysis, area-weighted (S/Si) grain size frequency distributions were obtained. Here, S represents the total area of the map and Si represents the area of the grains in the i*th* bin. To characterize the average statistical data of the structure, the volume fraction of HAGBs ($V_{HABs}$) was determined as the fraction of misorientations larger than 15° out of the total set of misorientations. Misorientations smaller than 2° were considered as integral misorientations, which occur as a consequence of the local curvature of the crystal lattice due to geometrically necessary dislocations (GND).

Precipitates in the ZK60 alloy were studied using the ThermoFisher Themis-Z (Eindhoven, The Netherlands) (200 kV) transmission electron microscope (TEM) with a field emission gun, equipped with high-angle annular dark-field imaging (HAADF-STEM detector) [27].

### 2.4. $H_{2}$-Evolution Test

To explore the corrosion behavior of the different states, the H_2_-evolution test was used in this study. For this test, a sample is immersed in an electrolyte. In the present case, the chosen electrolyte is phosphate-buffered saline (PBS) with a pH value of 7.4. PBS consists of 0.14 M NaCl, 2.7 mM KCl and 10 mM phosphate diluted in demineralized water. The test was carried out at room temperature. Due to the corrosion reaction of the sample, hydrogen is produced. The hydrogen is then caught in a special burette that has been modified with a funnel and is also filled with the electrolyte (similarly to [28]). This funnel ensures collecting all the hydrogen produced by the corrosion reaction. In the burette, the collected hydrogen replaces the electrolyte and, thus, the volume of the emerging hydrogen can be measured. The measurement of the hydrogen volume is carried out regularly during the test that takes seven to eight days in total. The obtained volume of hydrogen can be connected to the corrosion of the magnesium sample using the redox reaction of magnesium in aqueous environments [5]:

(1) $$Mg+2H_{2}O\to Mg{\left(OH\right)}_{2}+H_{2}$$

The redox reaction shows that one mole of emerging hydrogen results from the oxidization of one mole of magnesium.

### 2.5. Corrosion Structure Analysis

After the H_2_-evolution test, the emerging corrosion structure was investigated to collect more information about the type of corrosion. To preserve the corrosion build-up, the samples were also covered in cold mounting resin. Subsequently, cross-sections of assorted corroded samples were prepared using the same preparation method as described in Section 2.2.

Micrographs of the samples were taken using a Zeiss Axio Imager light optical microscope (LOM) (Oberkochen, Germany) at different states of the analyzing process: the first micrographs of the samples were taken of the polished surfaces with the bright-field setting before the corrosion tests. Using these micrographs and the corresponding information about the pixel sizes, ImageJ 2.9.0/1.53t was used to define the surface area of each sample that was submersed in the electrolyte. To define the pixels that belong to the sample surface, the image was firstly converted to an 8-bit image and then an individual threshold for each image was set to divide the image into white (sample) and black (background) pixels. By multiplying the number of white pixels with the corresponding pixel size, the submersed surface for each sample was defined.

After the H_2_-evolution test, the surfaces of the samples were looked at in the LOM. As the build-up of the corrosion products on some samples’ surfaces was protruding, Z-stack images of the samples were taken and stitched to get an overview of the surface. The images were taken using polarized light.

The cross-sections prepared from the assorted samples after the corrosion tests were investigated in the LOM using both bright-field and polarized light microscopy.

## 3. Results

### 3.1. Microstructure

**Figure 1:**
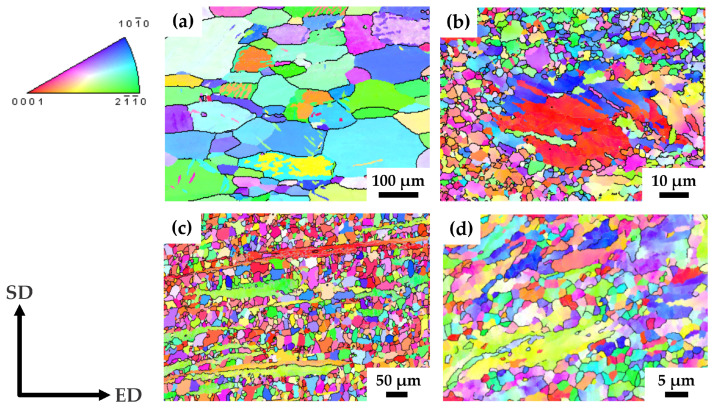
OIM maps from the longitudinal section of pMg-an (**a**), pMg-180 (**b**), ZK60-ex (**c**) and ZK60-150 (**d**) samples. SD and ED correspond to shear direction and extrusion direction, respectively.

The microstructure of the annealed and HPTE-processed pure magnesium is illustrated in Figure 1a and Figure 1b, respectively. The coarse grains in the annealed sample, with an average D15 size of 140 ± 1 µm (D2 = 127 ± 1 µm), have undergone a significant refinement to 1.7 ± 0.1 µm (1.4 ± 0.1 µm) as a result of the HPTE processing.

The majority of grains in the structure of the pMg-180 state are characterized by a diameter of 1–2 µm, excluding some coarse grains with a size of a few tens of micrometers (Figure 1b). These coarse grains contain twins, which indicates the unfavorable orientation of these grains for the development of the in-plane dislocation glide.

HPTE led to the microstructure refinement of the as-extruded ZK60 alloy as illustrated in Figure 1c,d. The mean grain size D15 in the as-extruded state was 28 ± 1 µm (D2 = 19 ± 1 µm), and the grains exhibited a notable extension along the ED axis in the longitudinal cross-section of the rod, as depicted in Figure 1c.

It was found that the grain size of the ZK60 alloy after the HPTE process is strongly dependent on the deformation temperature: D15 = 11 µm after HPTE at 250 °C and D15 = 1.6 µm after HPTE at 150 °C (Table 3). Figure 1d show the microstructure of the ZK60 alloy after HPTE at 150 °C. The microstructure mainly consists of small grains with a size of 1 µm; however, fragments of initially long fibers are still present in the structure. The length of these fibers is reduced to 20–50 µm. The results of the quantitative analysis of the microstructure for all sample states are collected in Table 3.

The phase compositions of the Mg-Zn-Zr tertiary alloys have been extensively studied [29,30]. Although the main hardening particles in Mg-Zn-Zr alloys are Mg-Zn intermetallic compounds, e.g., MgZn, MgZn_2_, MgZn_5_ and Mg_7_Zn_3_, fine precipitates containing Zr also form during hot deformation processing [31,32,33], which influence the recrystallization behavior and the microstructure of HPTE-processed samples.

**Figure 2:**
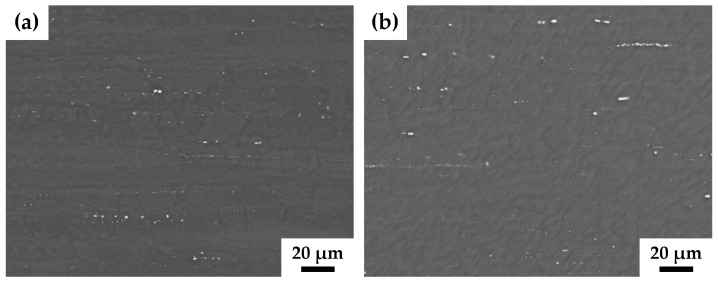
SEM-BSE images from the longitudinal section of the ZK60 alloy samples before (**a**) and after HPTE at 150 °C (**b**) with v6ω0.6 regime.

There are two types of particles in the alloy structure. The first type is large particles consisting of excess concentrations of zirconium. The maximum solubility of zirconium for the ZK60 alloy is 0.1% [31], while the alloy contains 0.19% zirconium (Table 1). It appeared that neither the size nor the spatial distribution of these particles were affected by HPTE processing. Large ZrZn particles form extended strings aligned along the ED direction in the structure of the alloy both after extrusion and after subsequent HPTE (Figure 2a,b).

**Figure 3:**
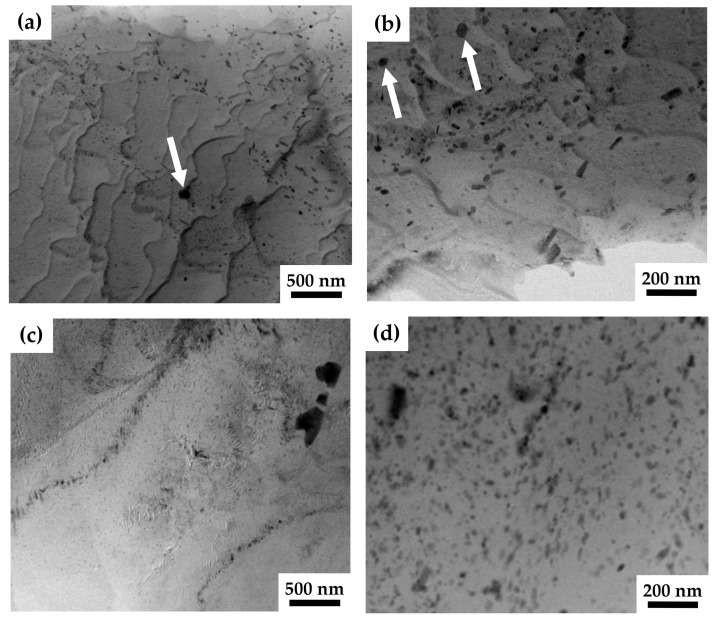
Bright-field (**a**–**c**) and HAADF (**d**) TEM images from the longitudinal section of the ZK60-ex (**a**,**b**) and ZK60-150 (**c**,**d**) samples. Globular particles are shown by white arrows.

Further analysis using TEM revealed that these coarse ZrZn particles were crushed during the HPTE process. In Figure 3c, the coarse particles have irregular shapes which resulted from their fracture during the HPTE deformation. TEM investigations revealed that, additionally to coarse ZnZr particles, fine particles were also present in the microstructure of the ZK60 alloy in both states, as-extruded and after HPTE. The bright-field images (Figure 3a,b) show the presence of two types of fine particles in the extruded sample, globular (shown with white arrows) and rod-like ones. These particles are very heterogeneously distributed in bands, which can be explained by the chemical inhomogeneity of the initial cast alloy.

According to the literature data and their typical morphology, these particles were identified as $\beta_{1}^{\prime }$-MgZn (Mg_7_Zn_3_) and Zn_2_Zr phases. The dark spherical particles highlighted with arrows in Figure 3a,b contain Zn and Zr. The elemental composition of such particles has been analyzed in Mg-Zn-Zr-alloys many times [30,31,34] and the presence of Zr and Zn in a Zn:Zr ratio close to 2:1 was confirmed. This is consistent with the expected Zn_2_Zr phase based on thermodynamic calculations.

The density of the secondary phase particles increased by 75% after HPTE at 150 °C in comparison to that in the as-extruded state (compare the HAADF image (Figure 3d) and bright-field image (Figure 3c)). Apparently, the complete precipitation of very fine Mg_7_Zn_3_ phase occurred, as expected, from the Mg-Zn-Zr phase equilibrium diagram at 150 °C [31].

### 3.2. Hydrogen Evolution

**Figure 4:**
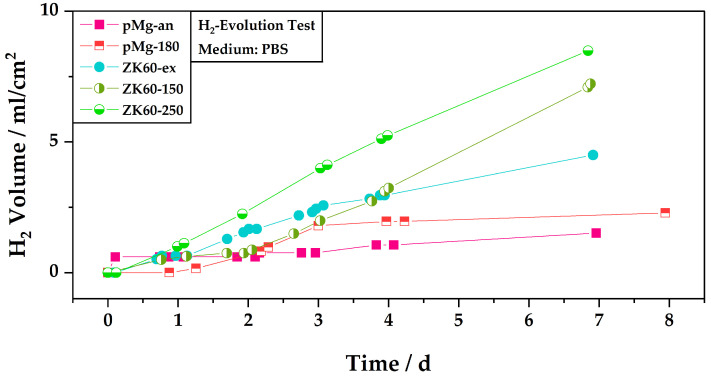
Overview of the hydrogen evolution of the different sample types during the immersion in PBS. The emerging hydrogen is normalized by the area of the sample that is exposed to the electrolyte.

Figure 4 displays the relative hydrogen volume generated by the corrosion reaction during the immersion tests in PBS for the five sample types. The volume of hydrogen evolved is normalized by the area of the respective sample’s surface exposed to the electrolyte so as to be able to compare the samples. The sample surfaces exposed to the electrolyte ranged from 0.3 cm^2^ to 0.4 cm^2^.

Figure 4 indicates that the corrosion activity of the samples is time-dependent. For example, the sample of pure magnesium in the annealed state (pMg-an) shows a steep increase in the hydrogen volume in the first hours and afterwards the evolution of hydrogen is rather slow. This is opposed to the sample pMg-180, which shows almost no increase in the hydrogen volume in the first day, then an increase in hydrogen volume in the following two days before the hydrogen volume stagnates again for the rest of the remaining test time. After two days, the hydrogen volume measured for sample pMg-180 is above the level of that of pMg-an.

At the beginning of the test, the levels of hydrogen evolution for sample ZK60-150 and sample pMg-180 are also comparable, which changes only after three days. Sample ZK60-150 shows a slower increase of hydrogen volume in the first two days as compared to the remaining test time, where the slope of the hydrogen evolution increases. Up to day four of the test, sample ZK60-150 also shows lower hydrogen volumes, and thus lower corrosion activity, than sample ZK60-ex. For ZK60-ex an inverse trend for the the hydrogen evolution compared to that of ZK60-150 can be observed. Here, the increase in hydrogen volume is quicker in the first three days before slowing down for the remainder of the test. The test for sample ZK60-250 shows higher hydrogen volumes as compared to all other samples almost from the beginning. Only the sample pMg-an with its steep slope in the beginning surpasses the levels for sample ZK60-250 in the first day.

When looking at the results after the end of the test, annealed pure magnesium shows the lowest rate of hydrogen evolution. The HPTE processed state of pure magnesium, with grain sizes up to 90 times smaller than the annealed state, leads to a higher volume of hydrogen.

At the end of the test the highest levels of hydrogen evolution are found for the ZK60 alloy, where, like pure magnesium, the application of the HPTE process leads to higher levels of corrosion and thus hydrogen evolution. Overall, the highest level of hydrogen evolution is displayed by the ZK60 sample treated by HPTE at 250 °C.

Comparing the change in corrosion activity for the different ZK60 states to their respective grain sizes, it becomes visible that in this study there is no direct link between grain size and corrosion activity. While the state with the biggest grain size, ZK60-ex (D2 = 19 ± 1), displays the lowest corrosion rate after seven days, the state with the lowest grain size, ZK60-150 (D2 = 1.3 ± 0.1), does not display the highest corrosion activity and even shows lower corrosion activity in the beginning. The highest corrosion activity is displayed by the state ZK60-250 (D2 = 2.7 ± 0.1).

Furthermore, the influence of the grain refinement on the corrosion activity is more pronounced for ZK60 than for pure magnesium. For pure magnesium, a decrease in the grain size of about 90 times leads to an increase in the generated hydrogen volume at the end of the test of about 51%, whereas for ZK60 a decrease in the grain size from the hot extruded state to the ZK60-150 state of about 15 times already leads to an increase in the hydrogen volume of 58%. The increase in the hydrogen volume is even more pronounced from ZK60-ex to ZK60-250 at 89% at a decrease of the grain size of about seven times.

However, it must also be kept in mind that in this study only one sample per state was investigated. As the corrosion activity is influenced by many variables, it is important to conduct these tests again with similar samples in order to obtain more data sets and statistical assurance.

### 3.3. Surfaces after Corrosion

**Figure 5:**
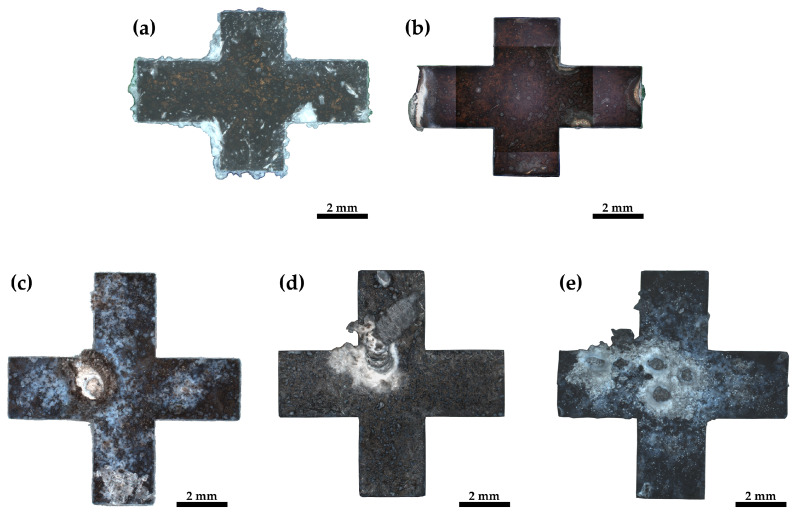
Light optical images of the surfaces of the different samples after immersion in PBS: (**a**) pMg-an, (**b**) pMg-180, (**c**) ZK60-ex, (**d**) ZK60-150 and (**e**) ZK60-250.

The surfaces of the immersed samples were looked at after corrosion tests in order to define differences in the corrosion mechanisms. In Figure 5, the surfaces of the five investigated samples are compared.

For the five samples in Figure 5, varying amounts of corrosion build-up can be seen. Correlating with the results from the hydrogen evolution measurement (Figure 4) the least build-up can be found for lower corrosion activities in pure magnesium and the most build-up is found for the ZK60 alloy, which displays the highest corrosion activity.

**Figure 6:**
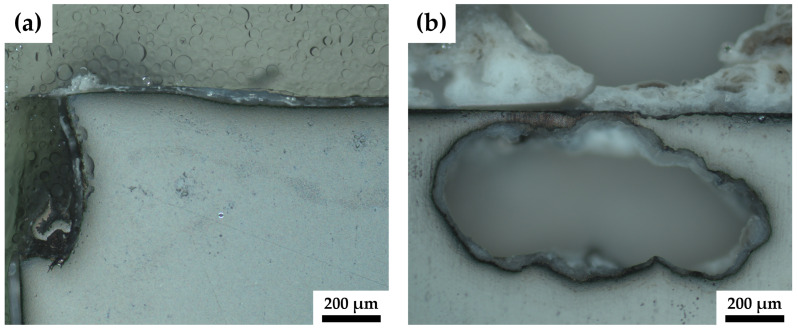
A close-up image of the crevice corrosion (**a**) of sample pMg-180 and of the pitting corrosion of ZK60-ex (**b**).

The pure magnesium samples, Figure 5a,b, show surface corrosion as well as crevice corrosion. Figure 6a depicts this crevice corrosion in further detail in a cross-section of the corroded sample. Crevice corrosion appears for pure magnesium at the border between the sample and the embedding material.

Besides surface corrosion, all ZK60 samples display pitting corrosion to varying degrees. The ZK60-ex sample, Figure 5c, shows surface corrosion and only one pitting corrosion hole and the surrounding build-up is rather small, whereas, for the sample of the HPTE 150 °C state, ZK60-150 (Figure 5d), two corrosion hot spots can be identified and the build-up for the larger one protrudes several millimeters from the surfaces. However, the amount of surface corrosion of this sample is rather small. Sample ZK60-250, depicted in Figure 5e, shows five pitting holes with much build-up and again more surface corrosion. As depicted in detail in Figure 6b for ZK60-ex, the pitting corrosion not only leads to holes that descend into the sample but also leads to corrosion that spreads laterally under the surface.

## 4. Discussion

A higher corrosion activity of ZK60 compared to the activities of pure magnesium, as depicted by the results at the end of the hydrogen evolution test, is expectable and reported in the literature [35,36,37]. Due to more alloying elements present in these ZK60 samples, the overall corrosion rate increases. In ZK60 magnesium is the most basic of the alloy elements which means that it is possible for micro-galvanic cells to form around second phase particles. This in turn can lead to a quicker and more localized corrosion of the sample, although it has been reported that Zn and Zr can also have positive effects on the corrosion activity of magnesium alloys [3,38,39]. Ding et al. [3] report that a content of Zn above 5% decreases the corrosion resistance. The ZK60 alloy used in this study, which exhibits the highest corrosion rates, contains 5.67% of Zn, which is just over the threshold of 5%.

The pitting corrosion observed for all investigated ZK60 samples is also linked to the second phase particles which show a more positive potential [40]. Besides the grain size and the percentage of alloying components, the second phase particles and their distribution play an important role in the corrosion behavior of magnesium alloys [4,41]. As mentioned before, second phases or segregations can lead to the formation of micro-galvanic cells which increase the corrosion activity of the magnesium matrix. The results of the microstructural analyses illustrated by Figure 2 and Figure 3 show that second phases of the more noble elements Zn and Zr are present throughout the samples. Even after the HPTE processing, relatively large secondary phase particles are still present; these segregations can act as micro-galvanic elements. This would explain why the corrosion behavior of the ZK60 elements is rather localized and little crevice corrosion could be detected.

The decrease in the corrosion activity for the pure magnesium samples, pMg-an and pMg-180, as well as the absence of pitting corrosion can be explained by the generation of Mg(OH)_2_ as described by Atrens et al. [17]. Mg(OH)_2_ has a low solubility in aqueous media and precipitates. It then increases the pH value locally and thus inhibits the corrosion activity. Zhao et al. describe how they measured a pH value of 10 in the layer close to the sample surface even though the immersion solution had a pH value of 4 [18]. This mechanism is also believed to be the reason for a lateral corrosion instead of a corrosion into the material, i.e., pitting corrosion [42].

It has been discussed in the literature that a grain size refinement can also lead to higher corrosion rates for magnesium and its alloys [10,13], although this can be highly dependent on the corrosion system comprising the metal alloy and the electrolyte [43]. The hypothesis that a grain refinement leads to a faster corrosion is also encouraged by the presented results. For the investigated samples of pure magnesium and ZK60 with different grain sizes, the alloys with smaller grains show higher corrosion activity when looking at the whole test time. Although considering the results for ZK60 as previously described, the correlation between grain size and corrosion activity is not simple. Looking at the results, where the biggest grain size leads to the lowest corrosion rate but the smallest grain size does not lead to the highest corrosion rate, other impacting factors must be taken into consideration. However, the results for the hydrogen evolution test presented in this study do show that the corrosion rates might change over the course of the immersion time. This time-dependent behavior depicted in Figure 4 shows the importance of longer immersion tests in order to define the overall corrosion behavior of magnesium alloys.

Furthermore, studies report that a higher dislocation density might also play a role in the increase in the corrosion activity of magnesium [4,16,44]. Comparing the results shown in Table 3, the dislocation density after HPTE processing is higher than in the initial states for both materials.

It is also known that residual stresses may affect the corrosion properties of magnesium and its alloys. For example, Denkena and Lukas [45] reported a significant improvement in the corrosion properties after surface treatment by deep rolling in the biocompatible Mg-3 wt.% Ca alloy due to a formation of compression residual stress. However, in the case of the present investigation, residual stresses may play a minor role, as the specimens with rather small dimensions were cut for the corrosion tests from the initial HPTE-processed rod, which most likely led to a significant release of these residual stresses.

The explanation for ZK60-250 having the highest corrosion activity although it does not have the smallest grain size or the highest dislocation density might lie in the processing temperature. The higher deformation temperature in the HPTE process initiates a higher growth rate of secondary precipitates, a larger volume fraction of small particles in the alloy and, accordingly, lower corrosion resistance.

## 5. Conclusions

In the present study, the microstructure of different magnesium alloys in different states was investigates. Subsequently, the five different magnesium sample types were immersed in PBS for seven to eight days. During the immersion test time, the emerging hydrogen was captured and its volume was regularly measured to obtain information about the corrosion activity of the different samples. After the corrosion test, the surfaces of the samples were looked at to define whether different corrosion mechanisms were observable.

Several conclusions can be drawn from these investigations:

1. The corrosion behavior of almost all tested samples as obtained from the hydrogen evolution test is time-dependent and shows different corrosion rates at different given times.
2. HPTE processing for both ZK60 and pure magnesium led to higher corrosion activity over the whole test time which was evident by the higher volume of hydrogen captured.
3. The corrosion behavior of the ZK60 states (ex, 150, 250) indicated further that a higher processing temperature at HPTE processing leads to even higher corrosion activity.
4. Pure magnesium showed surface and crevice corrosion, whereas the corrosion areas are more localized for ZK60. ZK60 shows pitting corrosion as the dominating corrosion mechanism. The localized corrosion behavior might again be due to the alloy elements that form segregations and thus micro-galvanic elements.

The results obtained in this study show again a strong connection between the microstructure of magnesium alloys and their corrosion properties, although no clear correlation between the grain size and the corrosion activity could be established. Furthermore, the results hint that the HPTE processing temperature might play an important role in the corrosion behavior of magnesium alloys. To verify this, further in-depth studies of the microstructure have to be carried out. In particular, possible segregations for differently processed alloys need to be investigated.

## Figures and Tables

**Table 1:** Chemical composition of Mg alloy ZK60. All values are weight %.

Alloy	Zn	Zr	Fe	Ni	Cu	Al	Mg
ZK60	5.67	0.19	0.0024	0.0006	0.0002	0.0030	Balance

**Table 2:** Material states investigated in the present study.

State	Material	Processing Route
pMg-an	pure magnesium	annealed at 500 °C for 30 min
pMg-180	pure magnesium	annealed at 500 °C for 30 min, extruded at 200 °C and HPTE 6v0.6ω at 180 °C
ZK60-ex	ZK60	extruded at 300 °C
ZK60-150	ZK60	extruded at 300 °C and HPTE 6v0.6ω at 150 °C
ZK60-250	ZK60	extruded at 300 °C and HPTE 6v0.6ω at 250 °C

**Table 3:** Structure parameters D2, D15, $V_{HABs}$ and GND.

State	D2/μm	D15/μm	$V_{\mathrm{HABs}}$/%	GND/ ×$10^{12}\;m^{-2}$
pMg-an	127 ± 1	140 ± 1	58 ± 1	24 ± 1
pMg-180	1.4 ± 0.1	1.7 ± 0.1	68.5 ± 0.5	507 ± 1
ZK60-ex	19 ± 1	28 ± 1	223 ± 1	114 ± 1
ZK60-150	1.3 ± 0.1	1.6 ± 0.1	50 ± 0.5	740 ± 1
ZK60-250	2.7 ± 0.1	11.0 ± 0.5	46.5 ± 0.5	465 ± 1

## Data Availability

The raw data supporting the conclusions of this article will be made available by the authors on request.

## Abbreviations

The following abbreviations are used in this manuscript:

EBSD	Electron Backscatter Diffraction
GND	Geometrically Necessary Dislocation
HAADF	High-Angle Annular Dark Field
HAB	High-Angle Boundary
HPTE	High-Pressure Torsion Extrusion
LAB	Low-Angle Boundary
LOM	Light Optical Microscopy
OIM	Orientation Imaging Microscopy
PBS	Phosphate Buffered Saline
SEM	Scanning Electron Microscope
SPD	Severe Plastic Deformation
TEM	Transmission Electron Microscope
UFG	Ultrafine Grained
